# Spatially Resolved Crosslinking of Hydroxypropyl Cellulose Esters for the Generation of Functional Surface-Attached Organogels

**DOI:** 10.3389/fchem.2019.00367

**Published:** 2019-05-24

**Authors:** Maximilian Nau, Simon Trosien, David Seelinger, Anna K. Boehm, Markus Biesalski

**Affiliations:** Laboratory of Macromolecular Chemistry and Paper Chemistry, Ernst-Berl Institute of Chemistry, Technische Universität Darmstadt, Darmstadt, Germany

**Keywords:** hydroxypropyl cellulose, surface modification, organo gels, surface patterning, photo-crosslinking

## Abstract

Chemistry, geometric shape and swelling behavior are the key parameters that determine any successful use of man-made polymeric networks (gels). While understanding of the swelling behavior of both water-swellable hydrogels and organogels that swell in organic solvents can be considered well-advanced with respect to fossil fuel-based polymer networks, the understanding, in particular, of wood-derived polymers in such a network architecture is still lacking. In this work, we focus on organogels derived from hydroxypropyl cellulose (HPC) ester. The latter polymer was functionalized with saturated and unsaturated fatty acids, respectively. Due to their tailored chemical constitution, we demonstrated that such polysaccharide can be crosslinked and simultaneously surface-bound by using a photo-induced radical reaction using a photo-initiator. Based on the choice of fatty acid used in the design of the HPC ester, and by controlling the degree of substitution (DS) obtained during the esterification of the polysaccharide, modular manipulation of the physical properties (e.g., polarity) of the resulting gel is possible. Depending on the initiator employed, different wavelengths of light, from UV to visible, can be utilized for the crosslinking reaction, which facilitates the deployment of a range of light sources and different lithographic methods. Additionally, we showed that altering of the illumination time allows to tailor the netpoint density, and thus, the degree of linear deformation in equilibrium and the swelling kinetics. Finally, we performed a proof-of-principle experiment to demonstrate the application of our material for the generation of spatially resolved polymer patches to enrich organic molecules from a solution within a microfluidic channel.

## Introduction

Swellable polymer networks have been extensively studied in recent decades, with subsequent development for use in a wide range of applications (Osada and Gong, 1998). In addition to hydrogels (networks that swell in water), which gained considerable attention during the last decades, because of their great potential in medicinal applications [e.g., tissue engineering (Annabi et al., 2014), drug delivery, and point-of-care diagnostics] (Rivest et al., 2007; van Tomme et al., 2008; Jagur-Grodzinski, 2009), organogels, which swell in organic solvents (Suzuki and Hanabusa, 2010), are highly interesting and offer promising perspectives in areas such as drug delivery (Vintiloiu and Leroux, 2008; Esposito et al., 2018), food applications (Marangoni, 2012; Chaves et al., 2018), cosmetics (Kirilov et al., 2014), separation, and purification processes (Venkatesan and Sarles, 2016; Lai et al., 2018; Prati et al., 2018), as well as analytics (Hinze et al., 1996; Mukhopadhyay et al., 2006; Xue et al., 2015). In general, the three-dimensional network structure of a polymer gel can be stabilized by various molecular interactions, in which the individual molecules are connected to each other by secondary forces [such as arene-arene interactions (Ajayaghosh and Praveen, 2007), halogen (Meazza et al., 2013), van der Waals forces, hydrogen bonds, and combinations thereof (Sangeetha and Maitra, 2005; George and Weiss, 2006; Hirst et al., 2008; Datta and Bhattacharya, 2015)] or covalent chemical bonds (Segarra-Maset et al., 2013; García et al., 2014). While adjusting the structure and the properties of gels is not trivial, when they are based on small molecules that are non-covalently connected, the design of covalently bound gels is considered more modular since various functionalities can be easily introduced, e.g., by the co-polymerization of functional groups or by the control of crosslinking density through the adjustment of potential cross-linking moieties in the gel constituting molecules. Furthermore, the use of covalently linked molecules implies highly stable materials whose macroscopic shapes are easily controllable when network formation is triggered by an external stimulus, e.g., by light (Hennink and van Nostrum, 2002). To create such a polymer network photochemically, one possibility is the reaction of double bonds in a radical reaction, e.g., by using a photo-initiator systems, such as camphor quinone/tertiary amine systems (Jakubiak et al., 2003), borates (Toba et al., 1997), or benzophenone derivatives (Merlin et al., 1980).

A variety of materials have been studied for the synthesis of such gels; among these, bio-based polymers such as cellulose and cellulose derivatives have been popular (Larsson et al., 2017; Wang and Zhang, 2018). Cellulose is a highly interesting compound because it provides a large number of valuable benefits: For example, as the most abundant polymer on earth, cellulose is highly available in bulk and originates from well-developed wood-disintegration processes (Klemm et al., 2005). However, because of the low solubility of unmodified cellulose in common organic solvents (i.e., alcohols, THF, chloroform, etc.), the controlled modification of the polysaccharide is highly challenging. Typically, harsh reaction conditions are necessary for heterogeneous functionalization, and the controlled partial substitution of the biomolecule is not trivial (Klemm et al., 2005). Therefore, various solvent systems have been established to improve a specific cellulose reaction, but all of these systems entail a range of challenges (e.g., toxicity, complex workup procedures, or limited scope of the chemical reactions; Heinze and Koschella, 2005). Due to this complexity, many scientists use cellulose derivatives rather than unmodified cellulose. Presently, a variety of cellulose-derived polymers are commercially available as bulk material. One important example is hydroxypropyl cellulose (HPC), which is readily available because of its industrial use in coatings and food applications (Wüstenberg, 2014). Like native cellulose, HPC provides 3 hydroxy groups per anhydroglucose unit (AGU) and exhibits a significantly higher solubility in many common organic solvents compared to the unmodified polysaccharide. Due to this property, further modification is technologically simplified, and the molecule can be, for example, esterified with various carboxylic acids in a one-step reaction while retaining good control of the degree of substitution (DS; Nau et al., 2018). Therefore, the polysaccharide can be easily equipped with various functions, such as fluorescence labels (e.g., pyrene; Winnik et al., 1987), that can be used as macro initiators for further polymerization (Ostmark et al., 2007), and control of the film formation properties and optical properties, amongst others, is possible (Bhadani and Gray, 1983). To use HPC as a base material for functional polymer gels, different approaches have been described in the literature. Gehrke et al. chemically crosslinked HPC by treatment with divinyl sulfone and analyzed the resulting microstructure and swelling characteristics (Harsh and Gehrke, 1991; Kabra et al., 1998). Other strategies that achieved covalent network formation of HPC include reaction with methacrylic anhydride (Hoo et al., 2013), *p*-formaldehyde (Suto, 1989), dialdehydes (Suto and Yoshinaka, 1993), and isocyanates (Suto et al., 1992), the formation of disulfide bonds (Tan et al., 2011), and gamma ray or electron beam irradiation (Wach et al., 2002). With respect to the light-induced formation of swellable HPC polymer gels, very few reports have been published to date. Bhadani and Gray esterified HPC with acryloyl chloride followed by photo-crosslinking already to stabilize the mesophase structure of a cholesteric film (Bhadani and Gray, 1984). The polymer was effectively crosslinked, and the structure was stable over a broad range of temperatures, but the mechanical properties of the resulting films were not optimal. An elegant approach was very recently described by Teramoto et al. (Yano et al., 2018). In their work, HPC was first esterified with cinnamoyl chloride, which can be crosslinked by illumination with UV light. The degree of crosslinking can be controlled either by adjusting the DS or by modulating the irradiation time. However, UV light (280 nm) is required for the crosslinking reaction, and relatively high DS values (1.3–3) are necessary for efficient network formation, which dramatically limits the flexibility of the system. Therefore, it is highly desirable to develop an alternative method that is more modular regarding polarity and light source.

In the present manuscript, we report a different approach for the photo-induced chemical crosslinking of HPC as films on solid model-surfaces using radical initiators. The approach is highly efficient and versatile with respect to the light source and thus allows for spatially resolved network formation in well-defined areas by using a commercially available 405 nm laser diode combined with an x/y movement system. In contrast to lithographic methods based on the use of photomasks (Böhm et al., 2013; Kargl et al., 2013), this method provides more versatile and faster approach tosurface patterning with less instrumental effort. In the first step, we functionalized HPC in a controlled fashion with unsaturated fatty acids, which can be crosslinked by using a photo-induced radical reaction. Via subsequent co-esterification steps, various functionalities can be introduced, yielding different and tailored functions of this polysaccharide. During the crosslinking process, *in situ* attachment of the polymer network to a model surface exposing allyl groups is possible. Consequently, we investigated the influence of the illumination time on network formation. For this purpose, the swelling process of the surface-bound gels was monitored via time-resolved confocal fluorescence microscopy. Finally, a microfluidic device was developed as a simple demonstration, in which a surface-attached functional HPC gel was used for the local upconcentration of organic model analytes, giving an interesting prospect for further research into low-instrumented sensing and/or purification devices.

## Materials and Methods

In this section, the most relevant results of the polymer synthesis are shown. For the clarity of the manuscript, further details for the syntheses and preparation procedures, a complete list of all solvents and reagents (including suppliers and purities), detailed information of all instruments and measurement methods, and details on reference experiments (bulk swelling) are shown in the Supplementary Material.

### Polymer Synthesis

**Figure 1:**
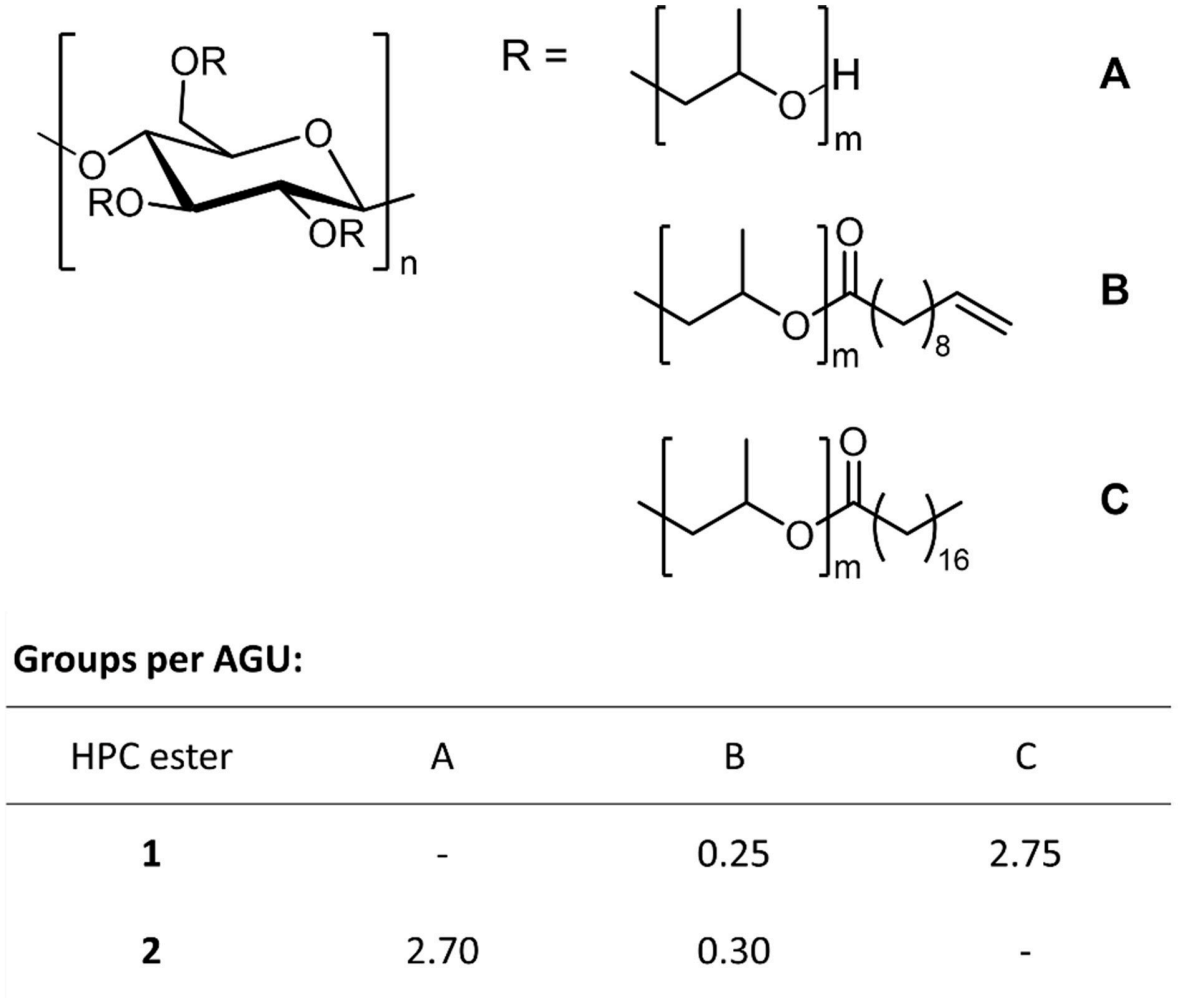
Synthesis of HPC esters **1** and **2**: Hydrophobic ester **1** exhibits 0.25 undecenoyl (B) and 2.75 stearoyl groups (C) per AGU, and hydrophilic ester **2** is esterified by 0.30 undecenoyl groups (B) per AGU, whereas 2.70 OH groups per AGU remain unmodified (A).

To synthesize the photo-crosslinkable polymers, we first dissolved HPC in THF (tetrahydrofuran) and then brought it to reaction with fatty acid chlorides. To generate a hydrophobic polymer, HPC was treated with stearoyl chloride (6 equiv.) and 10-undecenoyl chloride (1 equiv.) in a one-pot synthesis to afford a mixed HPC ester **1**. Details of the synthesis can be found in the Supplementary Material. The reaction led to degrees of substitution of 2.75 for stearoyl and 0.25 for undecenoyl moieties (DS were determined by NMR, see the Supplementary Material; Figure 1). In addition to this non-polar mixed ester, we synthesized a polar, hydrophilic polymer via the same method, which after cross-linking yielded a hydrogel rather than an organogel. To this end, we treated HPC with small amounts (0.5 equiv.) of 10 undecenoyl chloride so that hydrophilic reference ester **2** was obtained in quantitative yield, exhibiting a DS of 0.3 (Figure 1). Finally, the chemical structure of each polymer was characterized according to our recently published work (for details see Supplementary Material).

Note that stearoyl esters of cellulose are capable to form complexes with proteins such as bovine serum albumin (Niegelhell et al., 2017). For this reason, the formation of stearoyl esters, and tailored networks thereof is of particular interest.

## Results and Discussion

### Spatially Resolved Crosslinking and *in situ* Surface Attachment

Next, we were interested in designing organogels by attaching polymer **1** to the surface of a planar solid substrate in a spatially controlled fashion using a photo-initiator. Benzophenone derivatives, amongst others, are capable of generating radicals by illumination. These well-established photo-initiators provide various highly beneficial properties for the crosslinking of many systems: The activation and radical formation of benzophenone (BP) itself can be accomplished at relatively high wavelengths when compared to similar compounds (the upper absorption maximum of BP is located at λ = 340 nm; Allen et al., 1990). Consequently, it is not necessary to use high-energy light, which would otherwise be likely to damage sensitive molecules (Riga et al., 2017). Furthermore, radical formation with BP is a reversible process, implying high efficiency as an initiator (Dorman and Prestwich, 1994). If BP is functionalised with electron-donating or electron-withdrawing groups, the activation wavelength can be shifted, which allows the use of a broad range of light sources (Allen et al., 1990). To form the spatially controlled network of **1**, we decided to use a commercial laser diode (λ = 405 nm) as the light source because it is readily available and inexpensive. In addition, 405 nm light can be produced by light emitting diodes (LEDs), which opens the opportunity for the high-efficiency implementation of this method in a larger scale setting, in contrast to the use of mercury vapor lamps to generate UV radiation. Therefore, Michler's ketone [4,4′-bis(diethylamino)-benzophenone, DEABP] was found to be the most suitable initiator (see the Supplementary Material). To cross-link the reactive groups within the polysaccharide with functional groups on the solid substrate, appropriate pre-functionalization of the planar surface was carried out. We treated a glass slide with a mixture of allyltriethoxysilane (All-TES) and tetraethyl orthosilicate (TEOS; Ratio 15:85) by the method of Andrieu-Brunsen and co-workers (Krohm et al., 2016). Extensive characterization of this surface was reported in the literature (Krohm et al., 2016). Thus, a stable and homogeneous surface providing allyl functionalities was obtained. Note, allyl groups of All-TES are predestined for radical reactions and thus enable the polymer to be attached to the surface by a radical reaction pathway (Burkhard, 1950).

**Figure 2:**
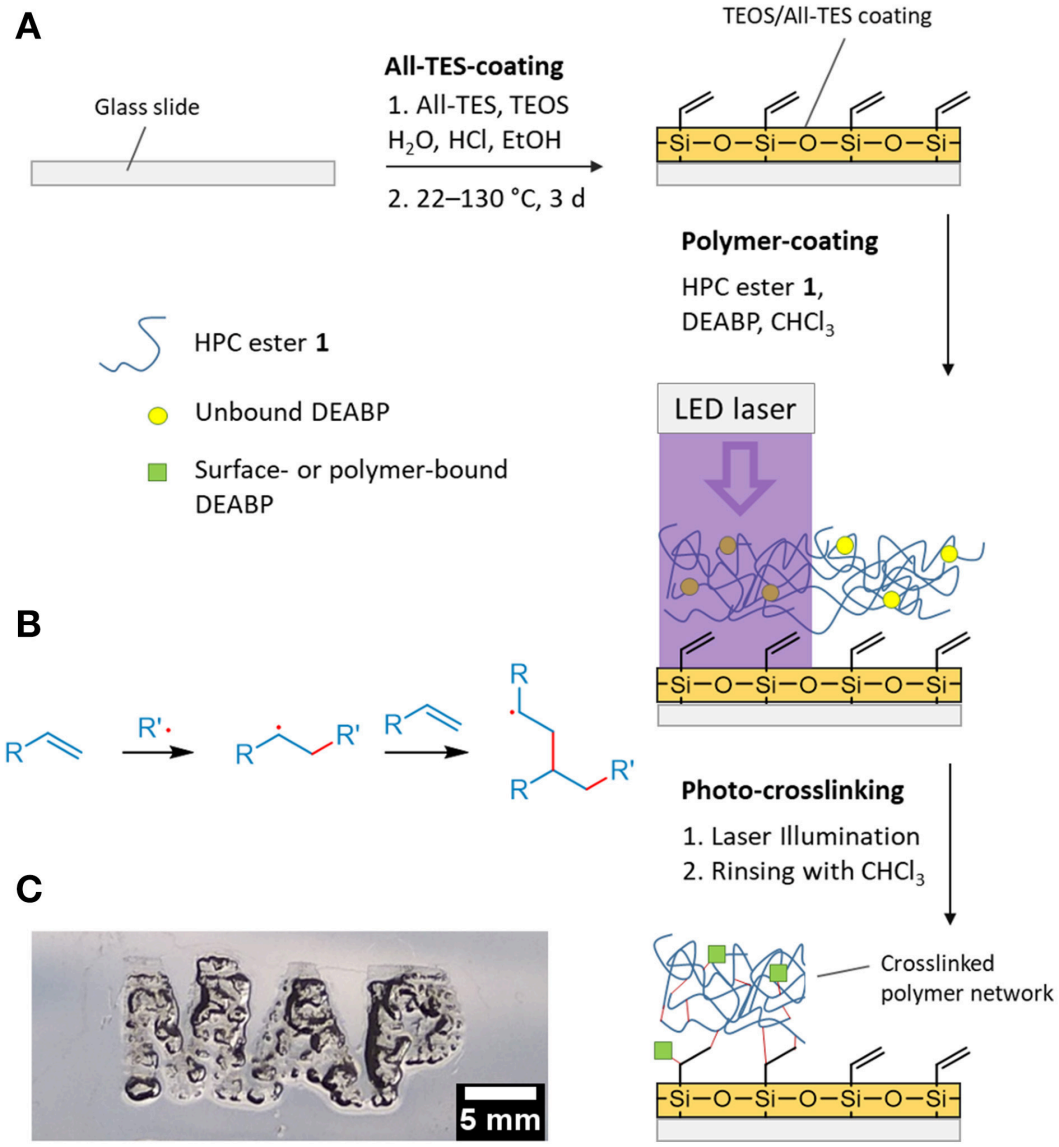
**(A)** Schematic of the production of surface-bound polymer network 1 crosslinked via laser treatment; **(B)** reaction scheme of the radical reaction between generated polymer radicals and double bonds of the polymer or the All-TES surface; **(C)** photograph of the surface-attached polymer network swollen in CHCl_3_. The illumination time used to generate the network was 20 ms with a 405 nm laser diode.

A DEABP-containing polymer solution in chloroform was solvent-casted onto the allyl-modified glass slide followed by air drying and treatment with short pulses of laser light (laser-diode, 1,000 mW, 100 μm spot dia., λ = 405 nm). The actual spatial resolution of this setup is currently limited by said spot diameter. A scheme of this process is shown in Figure 2A. By installing the laser in a computer numerically controlled (CNC) x/y movement system (see Supplementary Material), the production of polymer networks with well-defined geometries was easily accomplished (resolution: 350 DPI, limited by the control system; see Figure 2B). Finally, any unbound polymer was removed by rinsing with CHCl_3_, yielding a surface-bound swellable polymer network in well-defined regions (see Figure 2).

**Figure 3:**
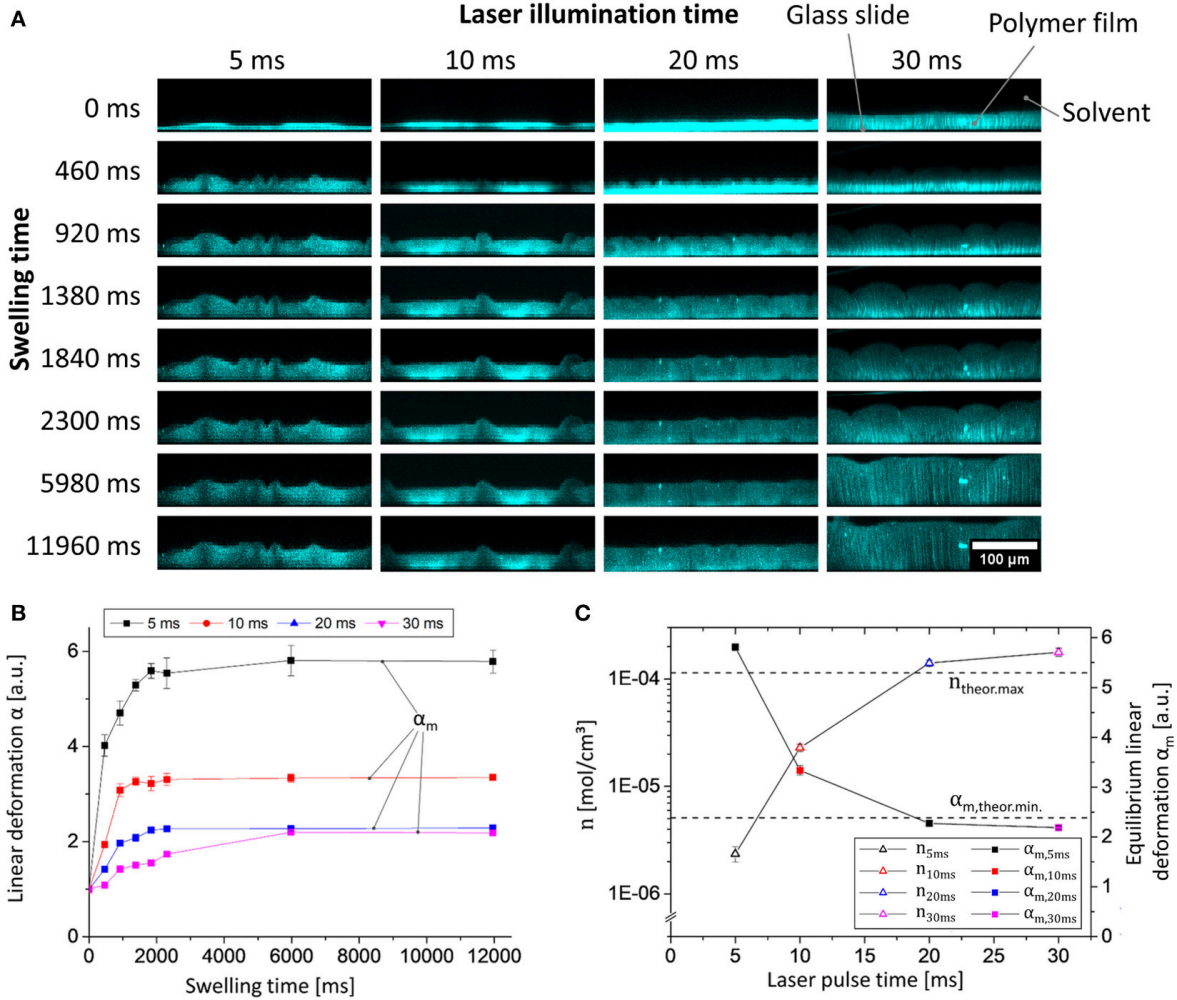
Data from experiments of the swelling of polymer **1** in chloroform photo-crosslinked by different laser illumination times (5, 10, 20, and 30 ms) measured via CLSM: **(A)** Still images of the dynamic x/z CLSM measurements (the microscopic morphology (i.e., non-flat) structure is the result of the spotwise laser illumination); **(B)** plot of linear deformation α vs. swelling time; **(C)** plot of linear deformation in equilibrium α_m_ vs. laser pulse time (blue plot); netpoint density calculated by equation 1 vs. laser pulse time (black plot) (theoretical maximum/minimum values are visualized as straight horizontal dashed lines).

Next, we examined the influence of the illumination time on the generation of the polymer network by laser light. To this end, the pulse-lengths during illumination were altered between 5 and 40 ms, and the swelling behavior of the spatially confined and surface-attached polymer networks was characterized by analyzing the linear deformation parameter α (one-dimensional relative swelling degree: the thickness of the swollen polymer film divided by the thickness of the dry polymer film) in equilibrium swelling. While α refers to the linear deformation in general we introduce α_m_ to as an additional parameter to denote α in equilibrium state. Therefore, the film thicknesses were determined by confocal laser scanning microscopy (CLSM; for details of the analysis see the Supplementary Material, for still frames captured at different times see Figure 3A). Note that DEABP itself is non-fluorescent when excited at a wavelength of 488 nm (the wavelength used in the CLSM measurements, see below) but becomes fluorescent after the crosslinking reaction due to alteration of the electronic structure of the molecule. The use of CLSM not only allows a static determination of the film thickness in equilibrium swelling but also enables dynamic monitoring of the swelling process (resolution: 460 ms; Figure 3B). For the comparison of the different samples, the parameter α is always referenced to their respective dry film thickness. By illuminating the deposited polymer film with laser light for 5 ms per spot, sufficient network points can be generated to form a surface-bound polymer network. If the illumination time is increased to approximately 30 ms, the degree of equilibrium linear deformation α_m_ of the polymer network exponentially decreases from α_m, 5ms_ = 5.8 to α_m, 30ms_ = 2.2, to a value that is in good agreement with the theoretical value of a quantitatively crosslinked polymer network in bulk (for details of the calculation of this particular value, see Supplementary Material; Figure 3C, square symbols). If the illumination time is further increased to 40 ms, the polymer network detaches from the surface. The latter phenomenon may be caused by decreased network flexibility, which leads to high mechanical forces and cohesive failure during swelling.

Because of their differences in chemical network structure, the individual samples show distinctly different swelling kinetics: If the netpoint density n is low, the diffusion of the solvent molecules to the substrate near the polymer layers is essentially unhindered by diffusion through the gel. Thus, the swelling behavior appears to follow first-order kinetics, as discussed for a similar system in the literature (Figure 3B, black line; Schott, 2006). If the netpoint density is increased, then the local viscosity increases, and the swelling kinetics becomes a product of the actual thermodynamically controlled swelling and diffusion processes, leading to a more complex behavior that may no longer be described by simple first-order kinetics (Figure 3B, purple line; Schott, 2006).

To learn more about the network structure from the swelling behavior, the netpoint density n of the surface-attached polymer gel can be estimated by using the Flory-Rhener equation. Because swelling is hindered by surface linkages, only one-dimensional swelling can occur. Consequently, to determine n, the linear deformation α_m_ may be considered instead of using the volumetric degree of swelling q_m_ (Toomey et al., 2004). Rühe and co-workers demonstrated that with surface-attached polymer networks, q_m_ scales as $\alpha_{\text{m}}^{9/5}$ and not as $\alpha_{\text{m}}^{3}$, as could be expected, because the surface-attached networks can only swell in one direction, i.e., away from the surface (Toomey et al., 2004). Finally, the Flory-Rhener equation can be transformed into equation 1 for the determination of the netpoint density of our polymer (for further details, see Supplementary Material).

(1) $$\begin{array}{r} \text{n}=\frac{-\left[\ln\left(1-\frac{1}{{\alpha_{\text{m}}}^{9/5}}\right)+\frac{1}{{\alpha_{\text{m}}}^{9/5}}+\chi {\left(\frac{1}{{\alpha_{\text{m}}}^{9/5}}\right)}^{2}\right]}{V_{1}(\sqrt[{3}]{\frac{1}{{\alpha_{\text{m}}}^{9/5}}}-\frac{1}{2{\alpha_{\text{m}}}^{9/5}})} \end{array}$$

In equation 1, V_1_ represents the molar volume of the solvent (i.e., 80.66 cm3/mol for chloroform), and χ is the characteristic Flory-Huggins polymer-solvent interaction parameter. In our case here, this parameter was estimated to be χ = 0.48 for 1 in chloroform at 22°C, according to incremental calculations by the method of Hoy (as shown in the Supplementary Material; van Krevelen and te Nijenhuis, 2009). As calculated from equation 1, the netpoint density of the polymer gel increases from n_5ms_ = 2.4 × 10^−6^ mol/cm3 to n_30ms_ = 1.8 × 10^−4^ mol/cm3 (Figure 3C, triangular symbols). Note that the latter apparently corresponds to the calculated maximum of n (n_theor.max_ = 1.1 × 10^−4^ mol/cm3, determined from the number of crosslinkable double bonds, see Supplementary Material) if the numerous approximations within the Flory-Rhener theory and Hoy's incremental calculation are taken into account (Valentín et al., 2008). Based on these findings, we are able to tailor the netpoint density to desired values simply by modifying the crosslinking parameters, without alteration of the polymer itself. This result demonstrates the versatility of our surface-attached cellulose-derived polymer network.

### Application in a Microfluidic Channel

**Figure 4:**
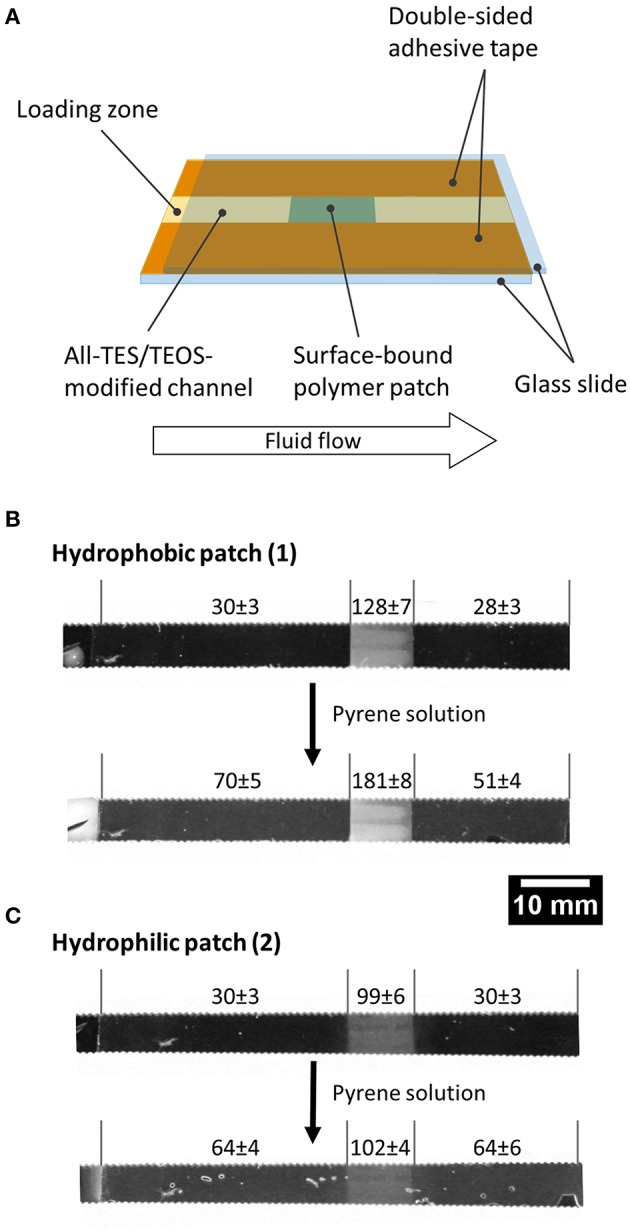
Microfluidic proof-of-principle device. A network of polymer **1** was generated within a microfluidic channel and used to collect pyrene from an aqueous pyrene solution: **(A)** scheme of the setup, **(B,C)** gray values of the channel before and after transport of the solution.

Because the prepared organogels can be attached to solid substrates, we were also interested in using the gels in a microfluidic demonstration device for the separation of an organic model pollutant from an aqueous solution. For this proof of principle a polymer patch, either made from polymer **1** or **2** was generated within a microfluidic channel comprising two glass slides held together by double-sided adhesive tape and with a capillary gap of 100 μm (Figure 4A). As a model substance for organic molecules (“pollutants”) in water, a saturated aqueous solution of pyrene was transported through the channel via capillary flow, and the gray values at different channel areas were determined during the experiment (Figure 4B; Supplementary Material). Note that the use of pyrene at the same time serves as a sensor due to its intrinsic fluorescence. If the solution comes into contact with the patch of crosslinked polymer **1**, pyrene accumulates within the patch to a very large extent, and the gray value (i.e., fluorescence) of the polymer patch increases as a result. Consequently, the mean gray value in the same area before the solution has passed through the patch is significantly higher than the value afterwards (Figure 4B), demonstrating the absorption and upconcentration of the pyrene in the patch. Note that pyrene did not accumulate in the channels that were composed of hydrophilic network patches of polymer **2**, because hydrophobic interactions are intrinsically prevented due to missing hydrophobic side chains as compared to the network composed of polymer **1** (see Figure 4C). Although the results reported here are qualitative, nonetheless, this simple experiment demonstrates that the surface-attached organogel is capable of upconcentrating non-polar organic molecules from solution.

## Conclusion

In conclusion, we developed a novel and versatile method to efficiently photo-crosslink hydroxypropyl cellulose derivatives for the generation of swellable polymer networks with a focus on organogels. The method is highly versatile and allows for the synthesis of organogels by esterification of the cellulose biomolecule with different fatty acids. By using DEABP as a photo-initiator, network formation can be induced by illumination with a conventional 405 nm laser [equipped with a computer numerically controlled (CNC) x/y movement system]. As a result, spatially resolved crosslinking of the polymer via laser illumination is possible. The swelling behavior (degree of swelling) and, thus, the netpoint density (estimated by the Flory-Rhener equilibrium swelling theory) of the surface-linked network, was determined via confocal fluorescence microscopy, yielding results that matched the theoretical estimation. This method allows the dynamic monitoring of the swelling process and, hence, the determination of the swelling kinetics. As a result, gels providing low netpoint densities swell through first-order kinetics, whereas the swelling of networks with higher crosslinking degrees follows a more complex mechanism. Finally, as a proof of principle, we generated a microfluidic channel based on a hydrophobic polymer network patch; our channel is able to concentrate an organic model dye within the patch, as opposed to a reference channel that is incorporating a hydrophilic polymer network patch. This is a promising starting point to increase the sensitivity of analytical devices, by locally increasing the analyte concentration. Via specific adjustment of the netpoint density, the control of the swelling behavior, and therefore, the flow speed and the dye-gel interaction, will be investigated in future trials.

## Author Contributions

All authors listed, have made substantial, direct, and intellectual contribution to the work. MN, DS, and AB performed all experiments. MN, MB, and ST analyzed the data, reviewed the literature, and wrote the manuscript. MB coordinated the complete project. All authors discussed and reviewed the results and approved the manuscript.

### Conflict of Interest Statement

The authors declare that the research was conducted in the absence of any commercial or financial relationships that could be construed as a potential conflict of interest.
