# Supplementary material for: Spatially Resolved Crosslinking of Hydroxypropyl Cellulose Esters for the Generation of Functional Surface-Attached Organogels
Source: Front Chem. 2019 May 24;7:367. doi: 10.3389/fchem.2019.00367 (PMC6543898; doi:10.3389/fchem.2019.00367)
Supplement: Supplementary file 1 [file Data_Sheet_1.PDF]

Supplementary Materials

Spatially resolved crosslinking of hydroxypropyl cellulose esters for the generation of functional surface-attached organogels

M. Nau, S. Trosien, D. Seelinger, A. Böhm, and M. Biesalski*

Laboratory of Macromolecular Chemistry and Paper Chemistry, Department of Chemistry, Technische Universität Darmstadt, 64287 Darmstadt, Germany

E-mail: biesalski@tu-darmstadt.de

Table of content

1. Materials, methods and instruments S-2
   a) Materials S-2
   b) Methods and instruments S-2
2. Synthesis and characterisation of the polymers S-4
3. Coating and crosslinking procedures S-6
4. Microfluidic experiment S-7
5. Determination of netpoint density n of crosslinked Polymer 1 by Flory-Rhener equilibrium swelling theory S-7
   a) Bulk polymer S-7
   b) Estimation of the Flory-Huggins polymer-solvent interaction parameter χ S-8
   c) Estimation of solubility parameter of polymer (δPolymer) estimated by the method of Hoy S-9
   d) Theoretical maximum value of the bulk polymer S-11
   e) Netpoint densities of surface-bound polymer 1 .... S-11
6. References S-11

1. Materials, methods and instruments

a) Materials

All reagents for synthesis were used of high purity. Grades and suppliers are listed in the table below:

Reagent	Comment	Supplier
Hydroxypropyl cellulose (HPC)	100.000 g/mol	Alfa Aesar
Stearic acid	97%	Merck KGaA
10-Undecenoyl chloride	97%	Sigma Aldrich
Sodium hydroxide	99%	Grüssing
Thionyl chloride	97%	Sigma Aldrich
Toluene	99.9%	Sigma Aldrich
Chloroform-D1	99.8%	Sigma Aldrich
n-hexane	Technical grade	Brenntag
2-propanol	Technical grade	Brenntag
$CaCl_2$	99%	Grüssing
Molecular sieve	3 Å	Roth
Tetrahydrofuran (THF)	98%	Roth
Chloroform	99%	Roth

Stearoyl chloride was prepared according to the procedure described in our prior work.[S1] Solvents were dried, if necessary, by using standard methods.

Double-sided adhesive tape was received from tesa® (NR. 56157).

b) Methods and instruments

Analysis of the microfluidic channel: The sample was positioned on a 365 nm UV-light source, and imaging was performed using a Cannon 700D with a Tamron SP 90 mm F/2,8 makro objective. The video data was analysed with ImageJ, where the color channels were separated and only the blue channel (where primarily the pyrene fluorescence is present) was used for grey-scale analysis.

Centrifugation was performed by using a Thermo Scientific Megafuge 8.

Dynamic CLSM (Confocal laser scanning microscopy) analysis of the swelling behavior was carried out using a Leica TCS SP8 CLSM. Fluorescence was excited with 488 nm OPS Laser, and fluorescence photons from 500 to 540 nm were selectively filtered for data acquisition and the pinhole was set yield 1 airy unit. The CLSM was operated in xzt-mode, which scans a single line (x) of the sample in z-direction at predetermined time intervals. The time interval was set to 460 ms as a compromise between resolution and acquisition time, i.e. if higher temporal resolution would be needed, lower spatial resolution would be achieved.

Errors for greyscale analysis of CLSM images and equilibrium swelling experiments are based on the standard deviation of multiple measurements and further determined through the Gaussian propagation of uncertainty.

Laser-crosslinking was performed by a self-made setup, consisting of a LED laser diode (405 nm, 1 W) and a x/y movement system. For obtaining well-defined light intensities, the setup was installed into an illumination chamber (see **Figure S-1**).

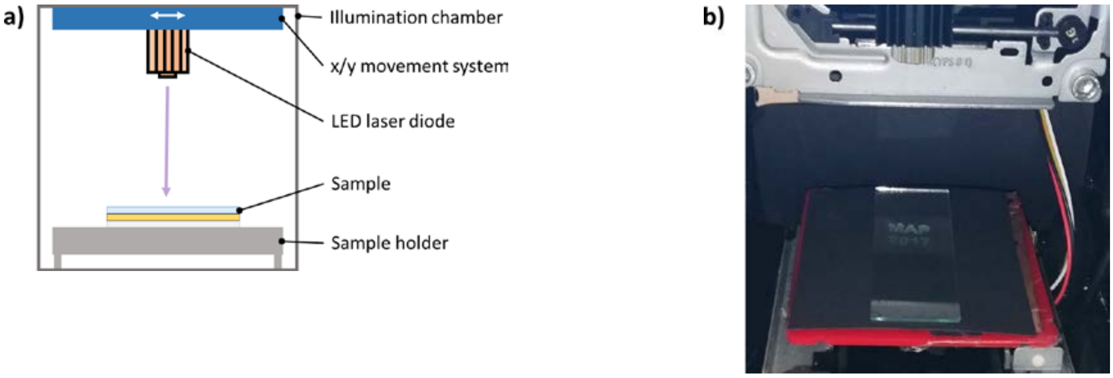

Figure S-1. Setup of illumination chamber used for laser crosslinking: a) schematic, b) photograph.

NMR spectra were recorded by using 300 MHz Avance III NMR and 500 MHz Avance DRX spectrometers from Bruker at 25 °C and analysed by MestreNova (MestreLab Research S.L.). ^{1}H spectra are reported in δ units, parts per million (ppm) downfield from TMS (tetramethyl silane) and are calibrated by reference to residual chloroform (7.26 ppm) in the deuterated solvent.[S2] Errors for ^{1}H NMR analysis were calculated via Gaussian error propagation with an assumed integral error of 5 %.

Photographs were captured with a Canon PowerShot SX220 HS (Canon Inc., Japan).

UV Photo-crosslinking was performed by using a 1000W Oriel flood exposure source (Newport, USA) equipped with an I-line filter at 365 nm.

UV/Vis spectra were measured by using a VARIAN CARY 50 spectrophotometer (Varian Inc., USA).

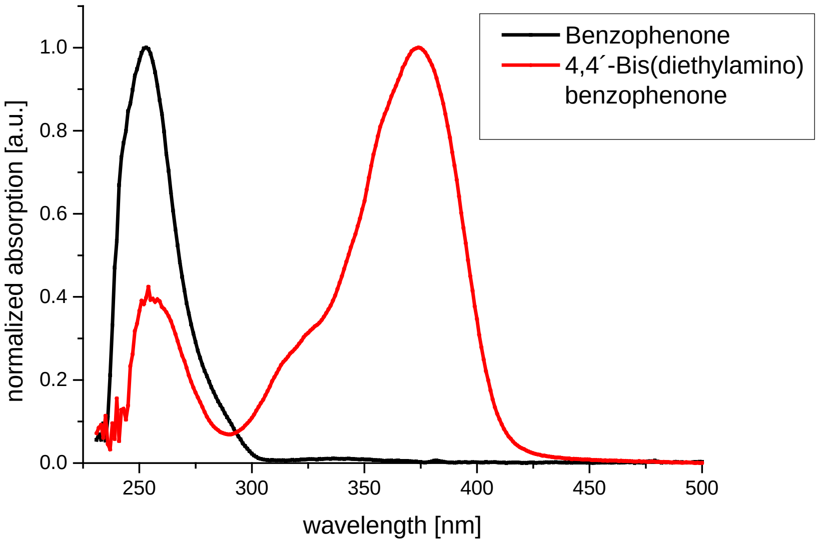

2. Synthesis and characterisation of the polymers

Synthesis of polymer 1

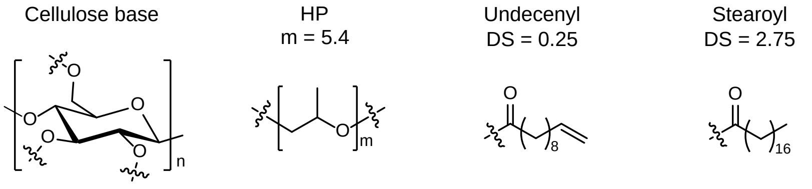

10 g (21 mmol, 1 eqiv.) of dried HPC was dissolved 200 mL dried THF. 4.24 g (21 mmol, 1 equiv.) of 10-undecenoyl chloride and 38.17 g stearic acid chloride were mixed with 120 mL dried THF in a separate beaker and added to the reaction mixture. This step is necessary since addition of the neat acid chlorides leads to precipitation due to a localised drop in solubility. The reaction was heated until reflux for 6 h, afterwards the reaction mixture was precipitated in four parts isopropanol, isolated via decanting, redissolved in THF and reprecipitated in isopropanol. This step was repeated four times, until finally the polymer was dried in vacuo for 48 h @ 40 °C. The final yield was 27.2 g (99%) with a DS of stearic acid of 2.75 and 0.25 of 10-undecenoyl acid respectively. The detailed analysis of the ^{1}H-NMR data for HPC fatty acid esters has been described in our prior work and has been performed accordingly, with the addition, that the multiplet at 5.8 ppm corresponds to one vinylic proton per 10-undecenoyl group.

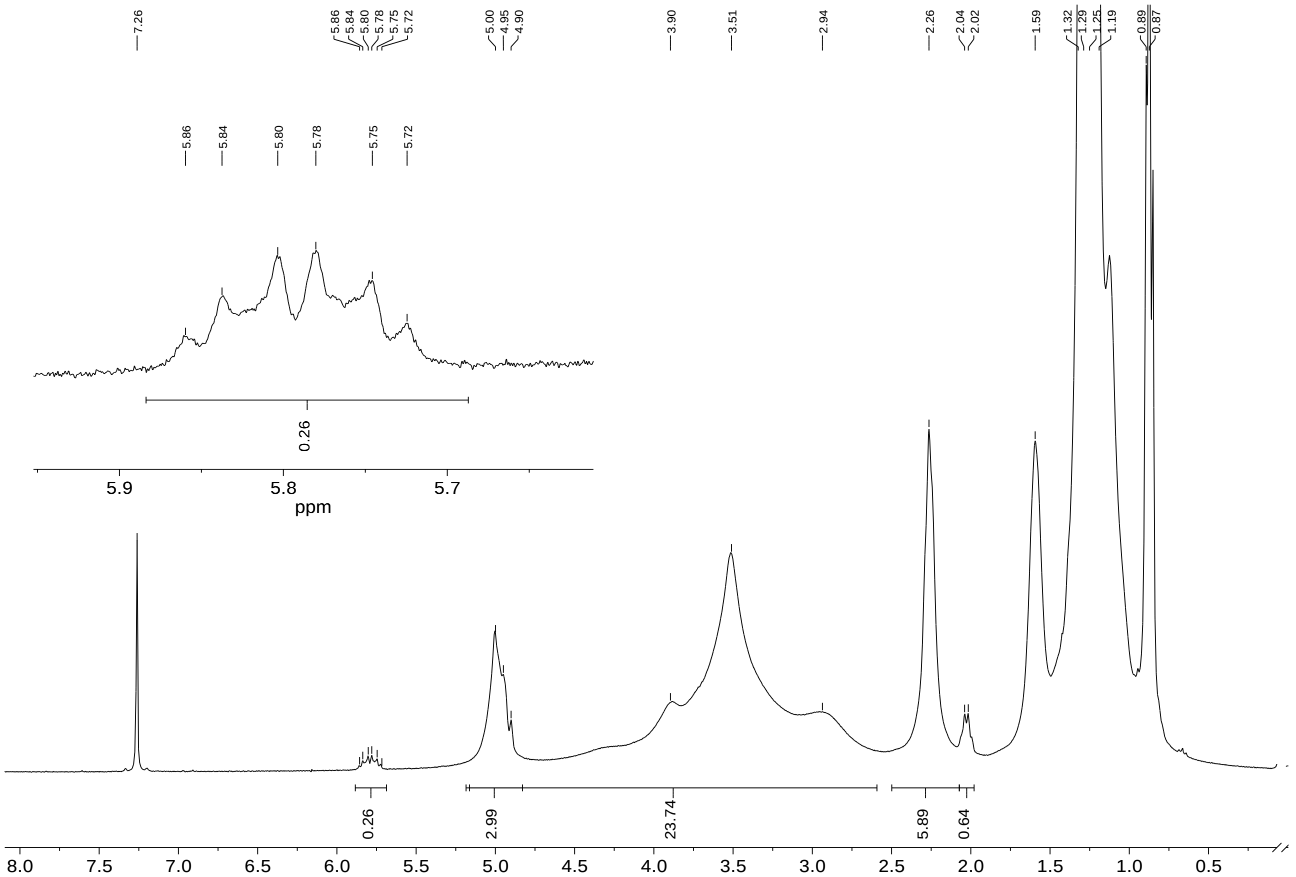

Synthesis of polymer 2

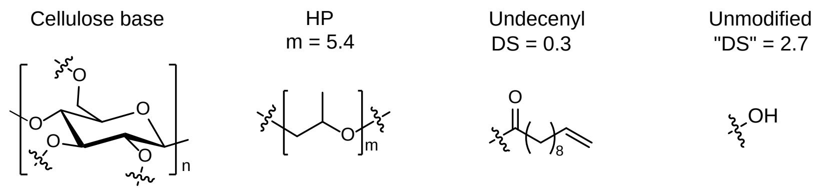

10 g (21 mmol, 1 eqiv.) of dried HPC was dissolved 200 mL dried THF. 2.12 g (10.5 mmol, 0.5 equiv.) of 10-undecenoyl chloride were mixed with 20 mL dried THF in a separate beaker and added to the reaction mixture. This step is necessary since addition of the neat acid chloride leads to precipitation due to a localised drop in solubility. The reaction was heated until reflux for 6 h, afterwards the reaction mixture was precipitated in four parts n-hexane, isolated via decanting, redissolved in THF and reprecipitated in n-hexane. This step was repeated four times, until finally the polymer was dried in vacuo for 48 h @ 40 °C. The final yield was 11.18 g (99.5%) with a DS of 0.30. The detailed analysis of the ^{1}H-NMR data for HPC fatty acid esters has been described in our prior work and has been performed accordingly, with the addition, that the multiplet at 5.8 ppm corresponds to one vinylic proton per 10-undecenoyl group.

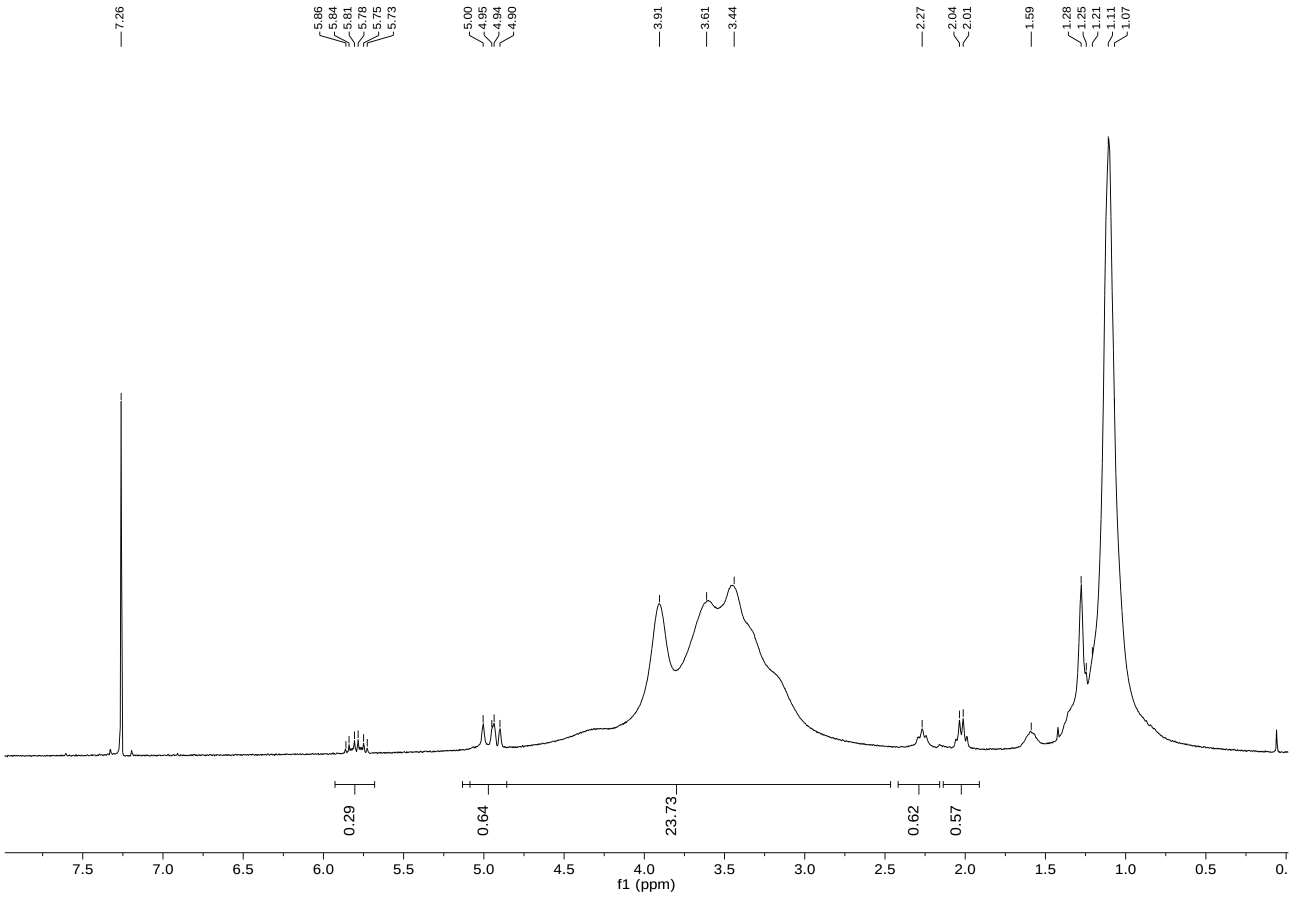

3. Coating and crosslinking procedures

Preparation of the All-TES/TEOS surface

A surface coating containing 85% TEOS and 15% All-TES was prepared and applied to a glass slides 76 mm * 26 mm (square) and 26 mm (round)) according to the protocol of Andrieu-Brunsen and co-workers.[S2]

Crosslinking and swelling experiments of the bulk polymer 1

Polymer 1 (300 mg) was dissolved in 5 mL chloroform and the desired amount of benzophenone was added. The solution was transferred to a petri dish and the solvent was evaporated in vacuo. Subsequently, the mixture was illuminated via UV light (λ=365 nm, UV dose=16 J/cm^2). The resulting polymer network was placed into a centrifuge tube (45 ml) and mixed by using a vortex mixing device for 15 s . Consequently, the solvent was removed via centrifugation followed by evaporation (22 °C, 30 min). After weighing of the gel mass, the polymer was dried again in vacuo (3 mbar, 40 °C, 20 h). To determine the degree of swelling q and the netpoint density n, the weights of the swollen and dried polymer network were used for calculation according to chapter 4a.

Preparation of the surface-attached network of polymer 1

A solution of 1 (1 ml, 25 mg/ml in $CHCl_3$) was treated with the desired amount of diethylamino benozophenone (DEAB). The solution was drop casted to the All-TES/TEOS surface coated round slides followed by evaporation of the solvent (22 °C, 1 bar → 20 mbar). Subsequently, the coated surface was illuminated by the laser diode and unbound polymer was removed by multiple swelling (8 h)/removing solvent (centrifugation, 3 min at 4000 rpm, 4500 G)/drying (3 mbar, 40 °C, 16 h) cycles (3 – 4 times). The degree of swelling (linear deformation α) and the netpoint density n was determined via CLSM according to chapter 4e.

Generation and use of the microfluidic channels

On a glass slide providing an All-TES/TEOS coating (Slide: 26 ×76 mm, coating: 15% All-TES, 85% TEOS), a 8 x 8 mm patch of desired polymer (**1** or **2**) produced according to the aforementioned procedure with a an illumination time of 30 ms was generated. Subsequently a channel of (76 × 6 mm) – incorporating the patch – was prepared through parallel sticking of two stripes of double-sided adhesive tape (Figure S-2). Finally, the channel was covered with a second glass slide retaining an opening at the head end to obtain the microfluidic channel that provide a “loading zone” (Figure S-2).

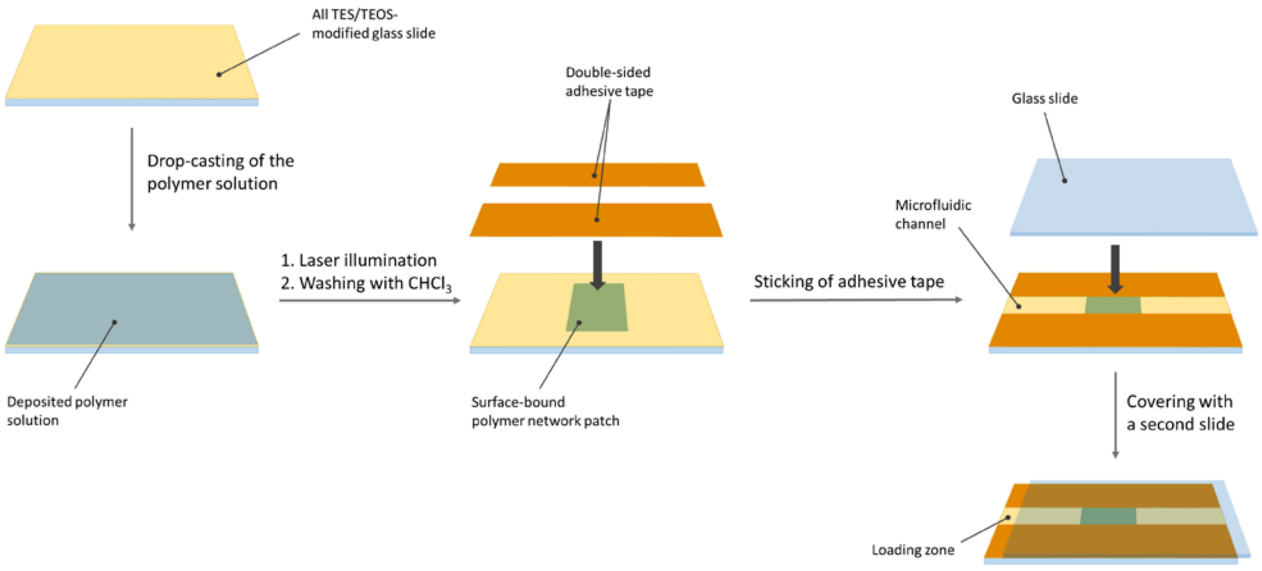

Figure S-2. Preparation protocol of the microfluidic channel providing a laser-crosslinked polymer network patch.

4. Microfluidic experiment

For the analysis of the up-concentration capabilities of the polymer patches made from **1** and **2**, a saturated aqueous solution of pyrene was applied to the loading zone of the microfluidic channel while it was placed under UV-illumination. The subsequent flow and up concentration was captured by the setup describes in section SI 1b. While the experiment with the hydrophobic polymer **1** shows a decrease in pyrene concentrations after the patch is passed – inferring up concentration in the patch – the hydrophilic polymer **2** (used as reference) doesn´t show a change in pyrene concentration, as displayed in Figure 4.

5. Determination of netpoint density n of crosslinked Polymer 1 by Flory-Rhener equilibrium swelling theory:[S4]

a) Bulk polymer

The netpoint density n was determined by (Equation S-1) that derives from the equilibrium swelling theory of Flory.[S4]

$$n = \frac{-[\ln(1-\nu_m) + \nu_m + \chi {\nu_m}^2]}{V_1\left(\sqrt[3]{\nu_m} - \frac{\nu_m}{2}\right)} \quad \textbf{(Equation S-1)}$$

V_1 represents the molar volume of the solvent, which is obtained from its molar mass (M) and its density (ρ) by $V_1 = M/\rho$ ($V_1 = 80.662\ cm^3/mol$). ν_m is the volume fraction of the polymer in gel, which can be calculated by the mass ratio of gel (X_r) and solvent (X_s) by using equation S-2. χ is the Flory-Huggins interaction parameter of the polymer and chloroform and is 0.48 (for detailed estimation see 4.a), Equation S-4).

$$\nu_m = \frac{V_o}{V} \quad \textbf{(Equation S-2)}$$

With V_0 = Volume of the unswollen polymer and V = volume of the swollen polymer. The volume of the unswollen polymer could be determined by its density and mass ($V_0 = m_0/\rho_{Polymer}$) (see Table S-1). V can be determined by the volume V_{solv} and the mass m_{solv} of the solvent, which are obtained by the gravimetrically determined masses of the swollen m_{gel} and the dried polymer m_0 as well as the density of the solvent ($\rho_{solv} = 1.48\ g/cm^3$) by $V_{solv} = m_{solv}/\rho_{solv}$ and $m_{solv} = m_{gel} - m_0$, by using (Equation S-3).

$$V = m_{gel} \cdot {\rho_{gel}}^{-1} = m_{gel} \cdot \left(\frac{V_0 + V_{solv}}{m_0 + m_{solv}}\right) \quad \textbf{(Equation S-3)}$$

Table S-1. Calculated values of the degree of equilibrium swelling q, the reciprocal degree of swelling ν_m and the netpoint density n of polymer **1** UV-crosslinked after addition of different amounts of benzophenone (BP) as initiator swollen in chloroform.

BP equiv.	yield [%]	q [a.u.]	ν_m [cm³/mol]	n [mol/cm³]
**0.005**	35 ± 2	11.401 ± 1.061	0.088 ± 0.008	1.22E-05 ± 2.7E-06
**0.01**	46 ± 3	9.472 ± 1.011	0.106 ± 0.011	1.92E-05 ± 5.0E-06
**0.025**	58 ± 3	9.386 ± 0.836	0.107 ± 0.009	1.96E-05 ± 4.3E-06
**0.05**	74 ± 3	7.322 ± 1.178	0.137 ± 0.022	4.35E-05 ± 1.5E-05
**0.1**	100 ± 6	5.955 ± 0.687	0.168 ± 0.019	8.16E-05 ± 1.9E-05
**0.2**	100 ± 5	5.694 ± 0.746	0.176 ± 0.023	9.39E-05 ± 2.5E-05
**0.5**	100 ± 6	6.050 ± 0.823	0.165 ± 0.022	7.77E-05 ± 2.2E-05
**0.8**	98 ± 7	6.782 ± 0.929	0.147 ± 0.020	5.48E-05 ± 1.6E-05
**1.0**	98 ± 7	6.477 ± 0.792	0.154 ± 0.019	6.30E-05 ± 1.6E-05
**1.2**	94 ± 9	6.566 ± 1.178	0.152 ± 0.027	6.04E-05 ± 2.3E-05

b) Estimation of the Flory-Huggins polymer-solvent interaction parameter χ:[S5]

General composition of polymer 1:

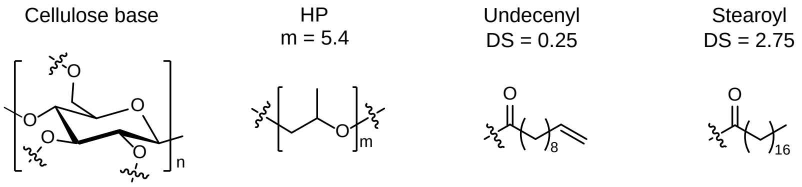

1 AGU = 1×Cellulose base + 3×HP(m = 5.4) + 2.75×Stearoyl + 0.25×Undecenyl

M_{AGU} = 2069 g/mol (molar mass of one AGU)

$\rho_{Polymer}$ = 0.94 g/cm³ (density of the polymer)

$$\chi = 0.34 + \frac{V_{Seg}}{RT}\left(\delta_{Polymer} - \delta_{Solvent}\right)^2 \quad \textbf{(Equation S-4)}$$

V_{Seg} = $M_{AGU}/\rho_{Polymer}$ = 2201 cm³/mol (volume of a polymer segment)

R = 8.31448 J/(molK) (ideal gas constant)

T = 293 K (temperature)

δ_{CHCl3} = 18.85 $(J/cm^3)^{1/2}$ (solubility parameter of chloroform from literature)[S6]

$\delta_{Polymer}$ = 18.45 $(J/cm^3)^{1/2}$ (solubility parameter of polymer, see 4.b), Equation S-5)

c) Calculation of solubility parameter of polymer (δPolymer) estimated by the method of Hoy:[S5]

$$\delta_{\mathrm{Polymer}} = \frac{\mathrm{F_t} + {}^{\mathrm{B}}/_{\mathrm{n}}}{\mathrm{V}_t} \qquad \textbf{(Equation S-5)}$$

B = 277 $(J/cm^3)^{1/2}/mol$ (base value)

n = 1

F_t = $\sum F_{t,i}$ (molar attraction function, for incremental contributions see **Table** S-2 and **Table S-3**)

= 32759.33 $(J/cm^3)^{1/2}/mol$

V_t = $\sum V_{t,i}$ (increm. mol. volume of the structural unit of the polymer, see **Table** S-2 and **Table S-3**)

= 1790.37 cm^3/mol

Table S-2. Values of increments for the molar attraction function of Hoy's System.[S5]

**Groups**	**$F_{t,i}$ [$(J/cm^3)^{1/2}/mol$]**	**$V_{t,i}$ [cm^3/mol]**
$-CH_2-$	269.0	15.55
>CH-	176.0	9.56
$-CH_3$	303.5	21.55
$=CH_2$	259	19.17
=CH-	249	13.18
-O- (ether)	235	6.45
-COO-	640.0	23.7
6-membered ring	−48	

Table S-3. Values of increments of substructures of polymer **1** calculated from the contributions of Table S-1.

**Groups**	**Number present in one AGU**	$F_{t,i}$ **[(J/cm³)^1/2/mol]**	$V_{t,i}$ **[cm³/mol]**
**Cellulose base**			
$-CH_2-$	1	269.0	15.55
>CH-	5	880.0	47.8
-O- (ether)	5	1175	32.25
6-membered ring	1	−48	
**Sum**		**2276**	**95.6**
**HP (m = 5.4)**			
$-CH_3$	5.4	1638.90	116.37
$-CH_2-$	5.4	1452.60	83.97
>CH-	5.4	950.40	51.624
-O-	4.4 (1 O is contributed to the ester groups)	1034.00	28.38
**Sum**		**5075.90**	**280.34**
**Stearoyl**			
$-CH_3$	1	303.50	21.55
$-CH_2-$	16	4304.00	248.8
-COO-	1	640.00	23.7
**Sum**		**5247.50**	**294.05**
**Undecenyl**			
$-CH_2-$	8	2152.00	124.4
$=CH_2$	1	259.00	19.17
=CH-	1	249.00	13.18
-COO-	1	640.00	23.7
**Sum**		**3300.00**	**180.45**
**Total**	$F_t = F_{t,\text{Cellulose base}} + 3\times F_{t,HP(5.4)} + 2.75\times F_{t,\text{Stearoyl}} + 0.25\times F_{t,\text{Undecenyl}} = 32759.33\ (J/cm^3)^{1/2}/mol$<br>$V_t = V_{t,\text{Cellulose base}} + 3\times V_{t,HP(5.4)} + 2.75\times V_{t,\text{Stearoyl}} + 0.25\times V_{t,\text{Undecenyl}} = 1790.37\ cm^3/mol$		

d) Theoretical maximum value of the bulk polymer

M_{AGU} = 2069 g/mol (molar mass of one AGU)

$\rho_{Polymer}$ = 0.94 g/cm³ (density of the polymer)

V_{Seg} = $M_{AGU}/\rho_{Polymer}$ = 2201 cm³/mol (volume of a polymer segment)

DS_{C11} = 0.25 (degree of substitution of unsaturated moieties)

Theoretically, each double bond can result in formation of 1 netpoints. With a $DS_{undecenyl}$ = 0.25, in 1 mol of polymer, 0.25 mol of bonds can be generated. Consequently, $n_{theoretically} = 0.25 \times 1/V_{seg}$ = 1.14*10⁻⁴ mol/cm³ can be generated in maximum.

e) Netpoint densities of surface-bound polymer 1

When $q = 1/\nu_m$ and 3-dimensional equilibrium swelling degree q_m is reduced to linear maximum deformation α_m ($\alpha_m = 1/\nu_m^{9/5}$),[S7] equation S-1 can be transformed to (Equation S-6) to determine the crosslink density n of surface-bound polymers during the swelling process.

$$n = \frac{-\left[\ln\left(1-\frac{1}{\alpha_m{}^{9/5}}\right)+\frac{1}{\alpha_m{}^{9/5}}+\chi\left(\frac{1}{\alpha_m{}^{9/5}}\right)^2\right]}{V_1\left(\sqrt[3]{\frac{1}{\alpha_m{}^{9/5}}}-\frac{1}{2\alpha_m{}^{9/5}}\right)} \qquad \textbf{(Equation S-6)}$$

Linear deformation is given by the film thickness of the swollen polymer L_m (in equilibrium) divided by the film thickness of the dried polymer L_0 and have been determined via fluorescence microscopy (see videos). For V_1 and χ the same values as in 3-dimensional swelling after equation 1 can be used ($V_1 = 80.662\ cm^3/mol$ and χ = 0.48). Linear deformation was determined via confocal laser scanning microscopy (see Figure 3a). Resulting values are listed in Table S-4.

Table S-4. Netpoint density n of surface-bound network of polymer **1**.

Illumination time [ms]	Linear deformation [a.u.]			n [mol/cm³]		
5	5.79	±	0.24	2,35E-06	±	3,79E-07
10	3.35	±	0.06	2,29E-05	±	1,84E-06
20	2.29	±	0.03	1,41E-04	±	9,42E-06
30	2.19	±	0.04	1,77E-04	±	1,56E-05
40	**Polymer delamination occurs**					

6. References

[S1] G. R. Fulmer, A. J. M. Miller, N. H. Sherden, H. E. Gottlieb, A. Nudelman, B. M. Stoltz, J. E. Bercaw, K. I. Goldberg, *Organometallics*, 2010, **29**, 2176-2179.

[S2] M. Nau, D. Seelinger and M. Biesalski, *Cellulose*, 2018, **134**, 44949.

[S3] P. J. Flory, Principles of Polymer Chemistry, Cornell University Press, Ithaca, New York, 1953.

[S4] F. Krohm, J. Kind, R. Savka, M. Alcaraz Janßen, D. Herold, H. Plenio, C. M. Thiele and A. Andrieu-Brunsen, *J. Mater. Chem. C*, 2016, **4**, 4067–4076.

[S5] D. W. van Krevelen and K. te Nijenhuis, Properties of Polymers, Elsevier, Amsterdam, Netherlands, 4th edn., 2009.

[S6] C. M. Hansen, *Ind. Eng. Chem. Prod. Res. Dev.*, 1969, **8**, 2–11.

[S7] R. Toomey, D. Freidank and J. Rühe, *Macromolecules*, 2004, **37**, 882–887.
